# A web-based self-help intervention for partners of cancer patients based on Acceptance and Commitment Therapy: a protocol of a randomized controlled trial

**DOI:** 10.1186/s12889-015-1656-y

**Published:** 2015-03-28

**Authors:** Nadine Köhle, Constance HC Drossaert, Karlein MG Schreurs, Mariët Hagedoorn, Irma M Verdonck-de Leeuw, Ernst T Bohlmeijer

**Affiliations:** Department of Psychology, Health and Technology, University of Twente, P.O. Box 217, 7500 AE Enschede, The Netherlands; Roessingh Research and Development, P.O. Box, 310 7500 AH Enschede, The Netherlands; Department of Health Sciences, University Medical Center Groningen and University of Groningen, P.O. Box 196, 9700 AD Groningen, The Netherlands; Department of Clinical Psychology, VU University, VdBoechorststraat 1, room 2B-64, 1082 BT Amsterdam, The Netherlands; Department of Otolaryngology/Head and Neck Surgery, VU University Medical Center, P.O Box 7057, 1007 MB Amsterdam, The Netherlands

**Keywords:** Cancer, Oncology, Distress, RCT, Partners, Self-help, Web-based, Effectiveness, Cost-effectiveness, Acceptance and Commitment Therapy

## Abstract

**Background:**

There is a growing recognition that cancer not only affects the lives of the patients, but also the lives of their partners. Partners of cancer patients are highly involved in the illness trajectory by providing informal care and they often experience distress. However, supporting interventions for this group are scarce and existing interventions bear several limitations. On the basis of the need for theory- and evidence-based supportive interventions for partners of cancer patients, the web-based self-help intervention *Hold on, for each other* has been developed. This intervention is based on Acceptance and Commitment Therapy. The primary objective of the RCT is to investigate the (cost-) effectiveness of the intervention. Additional goals are (1) to examine if psychological flexibility, self-compassion, mastery, supportive behavior, posttraumatic growth and resilience are mediators of the intervention’s effects on the partners’ mental health; (2) to examine the moderating effects of the socio demographics (age, gender, education, working situation, family situation) and disease-related characteristics of the patients (sort of cancer, stage of disease, duration and treatment of cancer); and (3) to investigate to what extend participants are satisfied with the intervention, which parts of the intervention are mostly used, and how adherent the users are.

**Methods/Design:**

A three-armed randomized controlled trial (RCT) will be conducted to compare two versions of the intervention *Hold on, for each other* with a waiting list control condition. Both intervention conditions contain the same content and differ only with regard to the form of professional support (personal support versus automatic support). Adult partners of cancer patients with mild to moderate depressive and anxiety symptoms, will be recruited through a multi-component strategy. Online measurements by self-assessment will be made on four measurement points (prior to randomization (baseline-measurement) and 3, 6 and 12 months after baseline).

**Discussion:**

When proven effective, *Hold on, for each other* can be an invaluable contribution to the healthcare system and it could be offered to all partners of cancer patients who are in need for additional support.

**Trial registration:**

Dutch Trial Register, trial registration number NTR4035, date of registration: 17 March 2013.

## Background

Being partner of a cancer patient is highly demanding. Partners are often involved in the illness trajectory by providing informal care and emotional support, and they regularly have to take on responsibilities of their ill spouse and the household, in addition to their own [1,2]. In recent years, there is growing recognition that cancer not only affects the lives of the patients, but also the lives of their loved ones. Recent studies have shown that partners and other family caregivers are at risk of experiencing mental and physical health complications. In a systematic review by Stenberg et al [3] 200 problems and burdens have been identified related to caregiving responsibilities among family caregivers. The most frequently reported problems were emotional (e.g. anxiety, depression and fear) and social problems (e.g. financial difficulties, role strain, isolation). Partners even suffered from diminished physical functioning and experienced complaints as pain, sleep problems and fatigue. Partners of cancer patients play an important role in patient recovery and illness management [3]. Therefore, the presence of mental and physical health complaints not only has a paramount impact on the partner’s quality of life, but it has also a negative impact on the informal care for the patient [4].

To overcome these problems, supportive interventions are available for partners of cancer patients. A recent meta-analysis [4] and two recent systematic reviews [5,2] identified a variety of psychosocial interventions for partners. However, most of these interventions were aimed at couples instead of the partner alone, and as a consequence the primary focus was often on the well-being of the patients. The needs of the partners have been overlooked and only a few interventions target the partners’ self-care as primary aim [4,5]. Another shortcoming of the existing interventions is that partners of cancer patients seem to make no or only limited use of them [6-8]. This might be a result of poor diffusion strategies or it might indicate that the interventions do not meet the needs and wishes of the target group. Finally, the interventions are often not theory-based and lack thorough evaluation [5,9]. Therefore, Ussher et al. [5] recommend high quality designs for future studies and better theoretical underpinning of the interventions to gain insight in the processes that might be relevant for partners of cancer patients.

The internet can be of added value in this respect, because it offers opportunities to deliver easy accessible and (cost-)effective interventions [4]. Advantages of web-based interventions for example are a low threshold and flexibility [10,11]. Participants do not have to make an appointment with a healthcare professional and they can use the web-based intervention at any moment or any location that suits them (24 hours a day, seven days in a week). This flexibility can be of great importance for partners of cancer patients, since they are often very occupied with caring tasks, and as a result have less time for their own health and personal activities [4]. Yet, despite these advantages, the web-based interventions for this population are scarce [9].

To overcome the above mentioned problems with existing interventions and to make use of the advantages the internet offers, we developed an online delivered, theory-based self-help intervention called *Hold on, for each other* to support partners of cancer patients. To make sure that the intervention fits to the needs and wishes of the end users, partners of cancer patients were actively and repeatedly involved during the developmental process. This paper presents the development of the *Hold on, for each other* intervention and the design of a randomized controlled trial to test the (cost-)effectiveness of this intervention.

### Theoretical framework

*Hold on, for each other* is based on Acceptance and Commitment Therapy (ACT). ACT is a form of contextual behavior therapy that focuses on changing a client’s relationship with their thoughts instead of changing the content of their thoughts [12]. Clients learn that avoidance, suppression or the attempt to control difficult thoughts can be counterproductive. They also learn to focus on behaviors and actions that are in line with their individual values – the things they care about most. ACT targets to increase psychological flexibility. Psychological flexibility is defined as “the ability to contact the present moment more fully as a conscious human being, and to change or persist in behavior when doing so serves valued ends” [13]. The efficacy of ACT in reducing psychological distress is supported by a growing body of literature (e.g. Hayes et al. [13], including studies among cancer patients [12,14]. ACT may be useful in partners of cancer patients, because it can help them to deal with the negative emotions caused by cancer (e.g. uncertainty, anxiety, sadness, anger) instead of avoiding these. Avoidance has been identified as one important factor resulting in psychological distress in cancer patients and their partners [15,16]. ACT may also help partners to cope with dysfunctional thoughts such as “what if the cancer comes back?” or “what if my partner dies?”. People are often excessively entangled with their thoughts and they need to learn to defuse from them [17]. This process of so called *cognitive defusion* or *meta- cognitive awareness* has already been proven to be effective in people with general anxiety disorder [18] and recurrent depression [19,20]. Finally, ACT may help partners of cancer patients to focus on what is really important to them (or in their relationship) and encourage them to act upon these values as much as possible, despite any barriers. This might especially be useful, as existing values, patterns and roles may have been seriously threatened or challenged by the cancer (e.g. Northouse et al. [4]; Applebaum and Breitbart [2]).

### Developmental process

To ensure that the intervention actually suits the partners’ needs and wishes, we used the method of co-creation, meaning that partners of cancer patients were actively involved during the developmental process and that their input was used to shape the content and the design of the intervention (see Table 1). First, we started with a needs assessment, consisting of an interview- and survey study. We interviewed 16 partners of cancer patients and asked them about their needs and wishes regarding the content and design of a web-based intervention and about the preconditions it should meet. During the interviews we also asked partners how their partner’s disease had affected them personally and what has been helpful to them to cope with the situation. We were interested in this information, because we wanted to gather examples of possible problems and solutions to write appealing and recognizable texts and exercises. To validate the results of our interview study, we also conducted a survey-study among 168 partners of cancer patients (results of both studies will be published elsewhere). The most important outcomes of both needs assessment studies were: (1) partners seem to be interested in a web-based intervention; (2) partners could spend about 1 hours a week on an intervention; (3) most prefer that at least some parts of an intervention are addressed to the partner alone; (4) the intervention should contain information and some form of peer support; (5) themes that should be addressed include coping with emotions, communication, sexuality, asking for help and moving on with life after cancer treatment; (6) partners differ in their preferences about the need for having contact with a personal counselor; (7) the intervention should be framed as informal, easy accessible support with a “positive approach” and (8) partners felt that flexibility is one of the most important features.

**Table 1:** **Developmental process of Hold on, for each other**

**Step**	**Aim**
1	What are the partners’ needs regarding a web-based intervention
	A) Interview-study
	B) Survey-study
2	Development of content material (texts and exercises)
3	Formative study: potential users are asked to evaluate content
4	Development of online application
5	Usability test and adaptation of the application
6	Effect study (RCT)
7	Economic evaluation

Based upon theoretical insights, consultations with experts and with the input from the interviews, texts were written and psychological exercises were prepared. At the end of this phase we had developed a first booklet version of the intervention *Hold on, for each other*. Next, we asked three potential users and one expert to evaluate the content. The participants were generally positive about the texts and exercises. They recognized the situations and examples given in the texts and they evaluated the exercises as useful. Yet, the participants also had some recommendations. For example, they suggested to provide more information on topics like sexuality and intimacy, financial and insurance issues and communication issues (e.g. how to communicate with younger children about the disease of their parent).

Based on their feedback, text materials were adjusted and the web-based application was developed. In a usability test, three partners of cancer patients and five immediate family members were observed while walking through the personal homepage of the intervention and the first module of the intervention. After using the intervention, they were asked to evaluate the web-based application. They found that the application was both useful and useable. Furthermore, they liked the conveniently arranged design, the use of fresh colors and the consequent structure of the different modules. The participants also made some suggestions to improve the web application. For example they said that some instructions of the exercises were unclear or confusing, they were not satisfied with the use of the colors of the “help”- and “home”-button and they disliked the image we had chosen as the header of the application. The participants’ feedback was used to improve the usability of the web-based application (for example a new header was implemented and the color of help-button changed from grey to red). At last, we will study the (cost-)effectiveness of the web-based intervention *Hold on, for each other* in a randomized controlled trial, that is described in this study protocol. Before we move on to the study questions, a short description of the intervention is provided below.

### Description of the intervention: ‘Hold on, for each other’

*Hold on, for each other* consists of six modules, which can be worked through in six weeks. In case participants need more time, they have the opportunity to work through the total intervention in maximal 12 weeks. In each module one particular theme is discussed. The first module focuses on the emotional consequences of being a partner of a cancer patient. Participants learn how to recognize, allow and express their emotions. In module 2, participants learn how to manage a period of chronic stress and module 3 focuses on worrying and negative thoughts. Module 4 and 5 are focused on values in life and in the relationship and the commitment to those values. Furthermore, the importance of beloved moments in a relationship are addressed. Module 6 is about the importance of communication. There are also two optional modules (participants can decide which is most relevant to them): one module concentrates on how to move on with life after successful cancer treatment; the other focuses on the terminal phase. If partners decide to do an optional module, they will receive two more extra weeks.

All modules start with a short text that matches the theme of each module (as described above). The texts are enriched with short psychological exercises. Both (texts and exercises) are based on ACT (a detailed overview of the components can be found in Table 2). Next to the ACT-exercises, in each module a meditation exercise (audio- and text-file) based on mindfulness and self-compassion is included. Mindfulness and self-compassion are congruent with ACT, and they can offer interesting insights to partners of cancer patients. Mindfulness can help partners of cancer patients to attend to the present moment in a receptive manner which will, over time, reduce the identification with self-focused thoughts and emotions that can lead to poorer mental health [21]. Besides, mindfulness can help partners of cancer patients to be aware of their painful feelings in a clear and balanced way. This means that they neither ignore nor exaggerate negative experiences [22]. Self-compassion is necessary to recharge batteries and emotional energy needed to care for others. Constantly criticizing oneself, especially for the feeling that one is never doing enough, will eventually lead to stress and symptoms of depression [23].

**Table 2:** **Modules, key components and example exercises of ‘Hold on, for each other’**

**Module**	**Key components**	**Example exercise**
1. Coping with your emotions	Acceptance	How I put on a brave face?: *Description:* We ask partners to write down emotional situations they have experienced, how they felt at that moment the situation occurred and how they coped with it. *Aim:* To help partners to be aware of their own emotions and their coping mechanisms. Are they regularly putting on a brave face and are they suppressing their emotions?
	Self-compassion/Mindulness	
2. Your resilience-plan – how can you keep going?	Acceptance	How much do you demand of yourself?: *Description*: We ask partners to write down how many hours they work, sleep, and have leisure time each week. *Aim*: To show partners how much they demand of themselves and if their planning is realistic.
	Self-compassion/Mindfulness	
3. My mind works overtime	Cognitive defusion	Worry Box: *Description*: In this exercise we ask partners to write down their thoughts, worries and fears on a piece of paper and put each paper in a box. After that they have to close the box and put it away. Later they can throw the box away or they can open it once in a month and read the worries again. *Aim*: To show that worries are often not based on firm grounds. The worry-box can help to put worries in perspective and it can show that ruminating is often useless.
	Self-compassion/Mindfulness	
4. What is now really important?	Values	Values in your relationship: *Description*: We asked partners to write down those things in their relationship that they value the most. *Aim*: To make them aware of things that are not congruent with their values. Are there things that should be different? Is it worth it to invest in the relationship? What can they do to come closer to their values.
	Self-compassion/Mindfulness	
5. Afraid, tired and moments of joy	Committed action	Celebrate your relationship: *Description*: we asked partners to choose activities (e.g. to write a love letter, to have dinner at their favourite restaurant). *Aim*: To make them aware of how precious their relationship is and how to live in accordance with their values.
	Self-compassion/Mindfulness	
6. The art of communication	Communicating about what really matters	What would you like to talk about?: *Description*: we ask partners to write down topics they have discussed lately with their partner, if there are topics that haven’t been discussed yet, and -if so- why these topics haven’t been discussed yet. *Aim*: To stimulate partners to communicate about the things that really matter.
	Self-compassion/ Mindfulness	
7. Moving on with life (optional)	Acceptance, cognitive defusion, values	Increase your hope: *Description*: We ask partners to imagine the situation that their partner is cancer free for almost a year, and that he/she is feeling alright. They -as a partner- have done everything possible to cope with the situation, they have accepted it and they are moving on with life. We ask them to imagine how life could be under these conditions. *Aim*: To show them that it sometimes can be helpful to create some distance and to have a closer look at their situation from a different point of view.
	Self-compassion/Mindfulness	
8. A good last period (optional)	Acceptance, communicating about what really matters, committed action	Beautiful memories: *Description*: We ask partners to think about (alone or with their spouse) what they can do to produce new memories (e.g. think about things you want to experience together, trips or activities you want to make). *Aim*: In this exercise a lot of aspects come together. To accept the development of the disease, to talk about what really matters at the moment, and to commit to values and live in accordance to them.
	Self-compassion/Mindfulness	

Next to information and exercises, participants also receive practical information, tips and references to relevant websites and organisations and the modules contain poems or inspiring texts. In order to optimally support the partners of cancer patients with completing the web-based intervention, three persuasive elements according to Kelders [24] were incorporated in the design of *Hold on, for each other*. First of all, the intervention contains a text message service. Participants can choose to receive text messages with short inspiring texts. Second, tunnelling is used in order to guide the participants through the intervention. And third, two types of social support are incorporated in the intervention: peer- to peer support and professional support. To facilitate peer–to-peer contact, participants have the possibility (1) to share their answers on some exercises with other participants (and to read those of others), (2) to add tips and advices and to read tips of others, and (3) to get in contact with other participants in a private e-mail conversation. If participants want to share their answers, or to contact other participants, they have to create a short profile first. This profile consists of: a (nick)name, sex, age, children and form of cancer the ill partner is diagnosed with. This profile provides partners with the opportunity to look for peers who are similar to them.

As mentioned before, the intervention also contains professional support. We want to investigate two different kinds of professional support: personal support (feedback on a weekly basis provided by a counsellor) versus automated support (feedback immediately after completing an exercise). Participants in the “personal support” condition receive weekly feedback from a counsellor through e-mail contact. After the completion of a module, a counsellor sends an e-mail to the participant (at an appointed day of the week) with a reflexion on the progress of the participant and a reaction to possible problems and questions. Participants have the same counsellor during the whole intervention period. Counselling is performed by trained master students Psychology of the University of Twente in the Netherlands who are under supervision of the researcher and a clinical psychologist. In sum, the role of the counsellor is the guidance and support of the process. Aim of the e-mail contact is predominantly to improve adherence of the intervention. In addition, participants developing serious problems during the enrolment in the intervention can be recognized and advised to find help. Participants in the “automated support” condition will receive short feedback messages directly after completing an exercise. The feedback is developed before the start of the intervention and the messages will appear in a pop-up window.

### Present study

This present study has several aims. First, we want to assess the (cost-)effectiveness of the intervention *Hold on, for each other*. Our main hypothesis is that both versions of the intervention lead to a significant reduction of psychological distress compared to a waiting list control condition. In addition, we hypothesize that (positive) mental health, health related quality of life and general health of participants of the experimental conditions will increase and caregiver burden will decrease in comparison to the participants in the waiting list control condition. The second aim of our study is to examine if psychological flexibility, self-compassion, mastery, style of support behavior (overprotection, protective buffering, active engagement), posttraumatic growth and resilience are mediators of the intervention’s effects on the partners’ mental health. Third, we aim to examine the moderating effects of the socio demographics (age, gender, education, working situation, family situation) and disease-related characteristics of the patients (sort of cancer, stage of disease, duration and treatment of cancer). Finally, we want to know to what extend participants are satisfied with the intervention, which parts of the intervention are mostly used, and how compliant the users are.

## Methods/Design

### Study design

This study is a prospective randomized controlled trial with three parallel groups:

1. Experimental condition 1: Web-based intervention *Hold on, for each other*, with personal support.
2. Experimental condition 2: Web-based intervention *Hold on, for each other*, with automated support.
3. Waiting list control condition: Participants will be on a waiting list for 3 months from entry/intake. They will receive Hold on, for each other (with automated feedback) after the first follow-up measurement, three months after the start of the intervention for the experimental condition.

This study has been approved by the Twente Medical Ethics Committee under the file number P13-17 (Dutch trial register: NTR4035). Participation is voluntary and all respondents will provide written informed consent before inclusion.

### Population and procedures

The population in this study consists of adult partners of cancer patients. To receive a heterogeneous group of participants, a multi-component recruitment strategy is followed (see Table 3). In all recruitment materials (e.g. advertisements, leaflets) the URL of the website (www.houvastvoorelkaar.nl) is mentioned, where respondents can find more information (including a short promotion video) and where they can apply to participate. On this website respondents can also read and download the patient information letter. Respondents can also do a self-assessment of eligibility on basis of the in- and exclusion criteria. Inclusion criteria are: (1) age of 18 years and older; (2) being partner of a cancer patient or cancer survivor; (3) having internet access; (4) no problems with the Dutch language; (5) and having mild to moderate symptoms of psychological distress symptoms (>3 on the Hospital Anxiety and Depression Scale (HADS) [25]. Exclusion criteria are: (1) severe anxiety (score on HADS-A ≥ 15) and severe depression (score on HADS-D ≥ 15); (2) recently started (less than three months ago) with psychological or psychopharmacological treatment; (3) not being able to spend 1-1.5 hours on the intervention every week; (4) partner died because of cancer and (5) diagnosis of partner’s disease is less than 3 months ago. Respondents who are eligible and would like to participate in the study can fill out an online contact form and will then receive an informed consent form by mail (reply envelope included). Respondents are asked to return the signed informed consent within a few days by mail.

**Table 3:** **Recruitment channels and recruitment strategies**

**Recruitment via**	**Recruitment strategy**
• National newspapers and magazines	Advertisements, newspaper articles
• Media	Interviews on radio and television, Twitter, Facebook
• Websites and magazines of relevant organizations (e.g. website of Dutch Cancer Society)	Online advertisements, newsletters
• Patient organizations and drop-in-centers	Online advertisements, newsletters, leaflets, presentations
• Hospitals and psycho-oncological organizations	Online advertisements, newsletters, leaflets, posters, presentations
• Other (e.g. psychologists, rehabilitation centers, general practitioners, physiotherapists)	Leaflets, posters

After receiving this informed consent, participants are sent an invitation by e-mail to fill out the HADS (14 items). People with severe anxiety and/or depression (cut-off score ≥ 15 on HADS-A and/or ≥ 15 HADS-D) [26] are excluded, because severe distress would require more intensive individual diagnostics and treatment. Participants that are excluded based upon severe psychological distress are contacted by telephone by a psychologist in order to be sure that these people are referred to adequate help. All partners with moderate scores on HADS-A and/or HADS-D (score 11-14 on HADS-A and/or HADS-D) are telephoned to assess the depressive episode module and the anxiety disorder modules of the Mini International Neuropsychiatric Interview (M.I.N.I.) [27]. In people screened as having a depressive disorder and/or an anxiety disorder by the MINI, the Sheehan Disability Scale (SDS) [28,29] is administered to measure the severity of their symptoms. Participants are asked to rate the extent to which work, social life and family life are impaired by their symptoms on a 10 point scale (0 = not at all impaired; 10 = extremely impaired). If participants report on at least two areas of their life severe impairment (scores ≥ 7) [28], they will be excluded from the study and will be strictly referred to seek adequate professional help. The telephone assessment of the M.I.N.I will be performed by trained and supervised master students.

### Randomization

**Figure 1:**
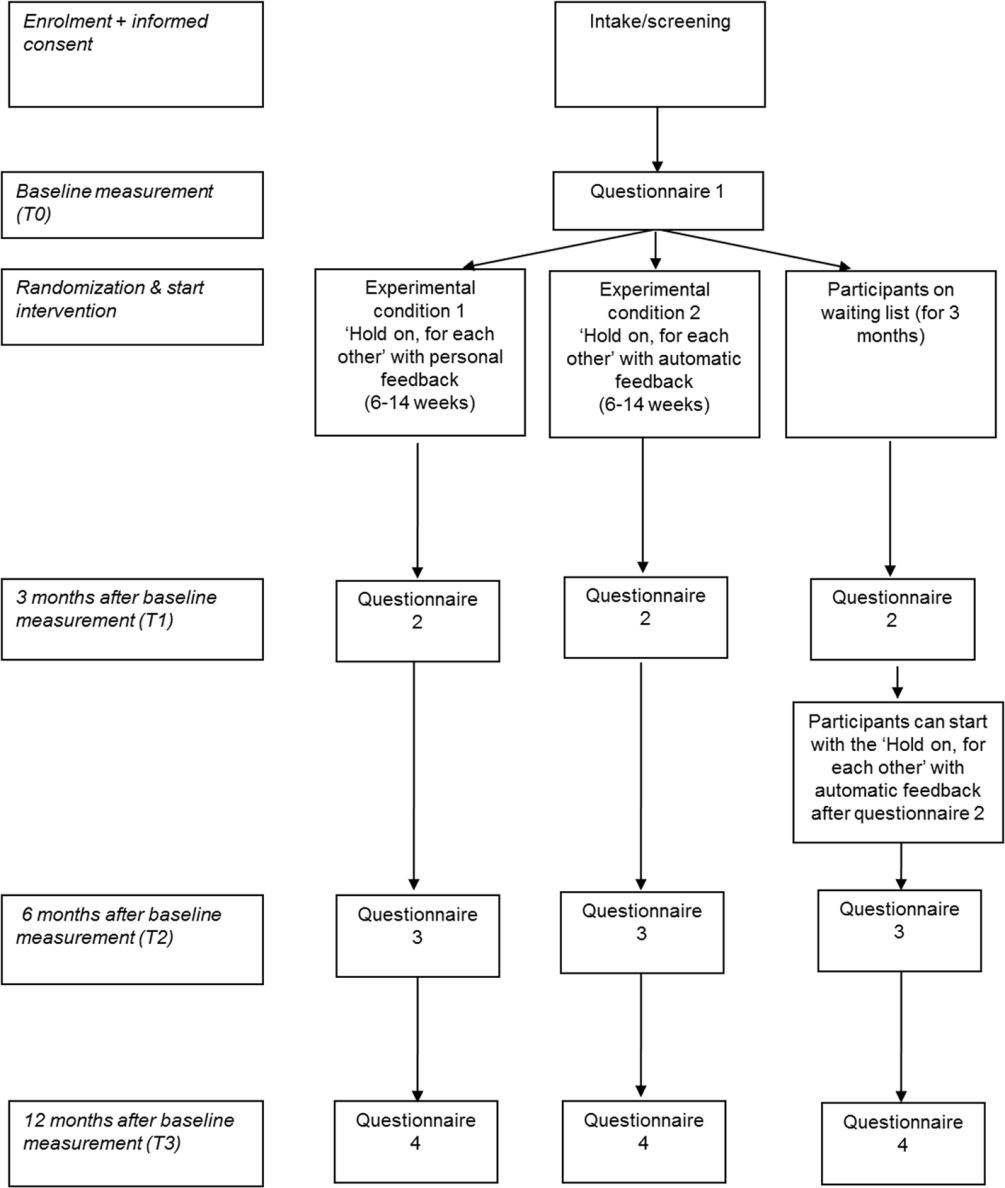
**Flowchart of ‘Hold on, for each other’.**

Respondents who are eligible, have provided their informed consent, and have completed the baseline (T0) questionnaire, are automatically randomized and divided over the three conditions. Randomization is stratified for gender, so an equal distribution of female and male participants in all conditions is warranted. Additionally we stratify for the perceived stage of disease, which is measured by the following item: (1) My partner is still in treatment and we have good hope that he/she will recover; (2) The treatment is completed and we are moving on with our lives; (3) My partner is unlikely to be cured. All participants receive an e-mail with the outcome of randomization and a link to start the intervention. Participants are informed about the three different conditions. They know that there is a waiting list control condition and that there are two experimental versions, one with automatic feedback and another with personal feedback. Participants in the experimental conditions will receive immediate access to the web-based intervention *Hold on, for each other*. The intervention is individually administered and the participants can access the intervention at any time, from any place, free of charge. Participants that will be placed on a waiting list have the opportunity to access treatment as usual (TAU) and they will be referred to relevant websites such as the website of Dutch Cancer Society (http://www.kwfkankerbestrijding.nl/ or http://www.kanker.nl) for information. Three months after the baseline measurement, which is directly after the first follow-up measurement at three months, participants on the waiting list receive the opportunity to follow the web-based intervention *Hold on, for each other* with automated feedback. They will also be invited to fill out measurements 6 and 12 months after the baseline measurement (see flowchart, Figure 1).

### Measurements

#### Overview

Table 4 gives an overview of all measurements. Participants will be asked to complete online questionnaires at baseline (T0); three months after the baseline measurement (T1); six months after the baseline measurement (T2); and twelve months after the baseline measurement (T3). The intervention as well as the questionnaires can be worked through in the participant’s own living environment.

**Table 4:** **Measurement overview**

		**Experimental conditions**				**Control condition**			
**Measure**	**Instrument**	**T0**	**T1**	**T2**	**T3**	**T0**	**T1**	**T2**	**T3**
*Socio-demographics and disease-related characteristics*									
Socio-demographics of the partner	Sex, age, education, marital status, cultural background, children, work status	X				X			
Disease-related variables of the patient	Sort of cancer, time of diagnosis, past and current treatment, current situation (prognosis)	X				X			
*Outcome measures*									
Psychological distress	HADS total	X	X	X	X	X	X	X	X
Mental health	MHC-SF	X	X	X	X	X	X	X	X
Caregiver strain	CSI	X	X	X	X	X	X	X	X
General health	RAND 36	X	X	X	X	X	X	X	X
Health-related quality of life	EuroQol EQ-5D								
*Mediators*									
Psychological flexibility	AAQ-II	X	X	X	X	X	X	X	X
Self-compassion	SCS-SF	X	X	X	X	X	X	X	X
Posttraumatic growth	PTGI-SF	X	X	X	X	X	X	X	X
Resilience	BRS	X	X	X	X	X	X	X	X
Sense of mastery	Pearlin Mastery Scale	X	X	X	X	X	X	X	X
Support behavior	Active engagement scale	X	X	X	X	X	X	X	X
*Evaluation*									
Client satisfaction	CSQ-8		X					X	
Evaluation form			X					X	
*Economic evaluation*									
Healthcare consumption	TiC-P	X			X	X			X
Production loss due to illness and absenteeism	PRODISQ	X			X	X			X
*Screening* ^*1*^									
Depression/anxiety (optional screening)	MINI (part: A, B, C, D, E, F, G, H, I, J, P)	X				X			
Severity of symptoms	SDS	X				X			

^1^The MINI and SDS will only be administered in people who have moderate scores on HADS-A and/or HADS-D (score 11 - 14 on HADS-A and/or HADS-D).

#### Socio-demographics and disease-related characteristics

The following socio-demographics of the partners are assessed: sex, age, education, marital status, cultural background, children, work status. Regarding their partner’s disease, participants are asked about type of cancer, time since diagnosis, current treatment and self-reported stage of disease (see description in paragraph ‘Randomization’).

#### Outcome measures

*Psychological distress*—the primary outcome—is measured with the total Hospital Anxiety and Depression Scale (HADS) [25]. The HADS is a 14-item questionnaire that measures the presence and severity of anxiety and depressive symptoms. Answering categories range from 0 to 3 and the items are added to a scale score that can range from 0 – 42. Higher scores mean more symptoms of psychological distress.

*Mental health* is assessed with the Mental health Continuum – Short Form (MHC-SF) [30,31]. The MHC-SF is a 14-item questionnaire that measures three dimension of positive mental health [30]: emotional well-being (3 items), psychological well-being (6 items), and social well-being (5 items). Participants are asked to rate the frequency of feelings they have experienced in the past month. Items are scored on a 6-point scale ranging from 1 to 6. A mean score is computed ranging from 1 to 6 with higher scores indicating higher levels of positive mental health. The Dutch version of the MHC-SF has shown good psychometric properties [31].

*Caregiver strain* is assessed with the 13-item Caregiver Strain Index (CSI) [32]. The CSI contains at least one item to measure burden in each of the following major domains: employment, financial, physical, social and time. Each item can be answered with ‘yes’ (1) or ‘no’ (0). A CSI score is computed by counting the number of ‘yes’, resulting in a score from 0-13. The CSI has shown good psychometric properties [32].

*General health* of the partners of cancer patients is assessed with one item of the RAND 36 [33,34]. This item “How would you rate your own general health?” can be answered on a 5-point scale ranging from 1 - 5, with higher scores indicating better general health.

*Health-related quality of life* of the participants is measured with the EuroQol EQ-5D [35]. The EQ-5D is a validated instrument for measuring health-related quality of life and it covers five domains of health: mobility, self-care, usual activity, pain/discomfort and depression/anxiety. Each of the five domains has three severity levels; 0 (none), 1 (some), and 2 (severe).

#### Moderators and mediators

In this study age, gender, stage of disease and compliance (frequency and time spend on the website) are examined as moderators. The following variables are examined as mediators: psychological flexibility, self-compassion, posttraumatic growth, resilience, styles of support behavior and sense of mastery.

*Psychological flexibility* is measured with the 7-item Acceptance and Action Questionnaire II (AAQ-II) [36,37]. Answering categories range from 1 to 7 and the items are added to a scale score that can range from 7 to 49, with higher scores indicating more psychological flexibility [36]. The Dutch version of the AAQ-II has shown good psychometric properties [38] also in adults with psychological distress [39].

*Self-compassion* is measured with the Self-compassion Scale Short-Form (SCS-SF) [40,41]. This questionnaire consists of 12 items and is measuring six components of self-compassion: self-kindness, self-judgment, common humanity, isolation, mindfulness and over-identification (2 items for each component). Respondents rate on a 7-point scale ranging from 1 to 7. A mean score is computed with higher scores indicating higher levels of self-compassion. The SCS-SF has adequate psychometric properties [41].

*Posttraumatic growth* is assessed with the 10-item Posttraumatic Growth Inventory- Short Form (PTGI-SF) [42,43]. All items are positively formulated and comprising five factors: (1) relation to others (2 items); (2) new possibilities (2 items); (3) personal strength (2 items); (4) spiritual change (2 items); and (5) appreciation of life (2 items). Respondents rate each item on a 6- point scale ranging from 0 to 5 and the items are added to a scale score that can range from 0 - 50, higher scores indicating higher posttraumatic growth. The questionnaire has shown good psychometric properties [43].

*Resilience* is measured with the 6-item Brief Resilience Scale (BRS) [44]. The BRS assesses the ability to bounce back or recover from stress. Respondents rate on a 5-point scale ranging from 1 to 5. The BRS is scored by averaging the scores on the items (after reversing the negative ones). The score can range from 1 to 5, higher scores indicating higher levels of resilience. The BRS has shown good psychometric properties [44].

*The different styles of support behavior* are assessed with the 19-item Active engagement scale [45]. Five items constitute the active engagement scale, 8 items measure protective buffering and 6 items measure overprotection. Items can be answered on a 5-point scale ranging from 1 to 5. For each subscale, the items are averaged within subjects into a scale score that ranges from 1 - 5. The Active engagement scale has shown good psychometric properties [46].

*Sense of mastery* is measured with the 5-item Pearlin Mastery Scale (PMS) [47]. The instrument measures the extent to which one regards one’s life chances as being under one’s own control in contrast to being fatalistically ruled. Items are scored on a 5-point scale from 1 to 5. The scores on the items are added to a sum score that can vary from 5 to 25. High scores signify that the individual perceives him or herself in control of his or her life.

#### Evaluation of the intervention

To measure the client satisfaction after the intervention, the 8-item Client Satisfaction Questionnaire (CSQ-8) [48] is used. All items are scored on a 4-point scale ranging from 1 to 4. For each individual a ‘satisfaction-score’ is computed by averaging their scores on the 8 items. Also a question is included about how the participants evaluated the intervention on a scale from 1 to 10. Furthermore, the participants are asked (1) the amount of hours they have on average spend completing the intervention; (2) to what extent they have completed the exercises; (3) if they have used the different components of the web-based intervention; and (4) if they were satisfied with the received feedback. We also asked them to write down three aspects of the intervention they appreciated most and three things they appreciated least. Finally we asked them if they have suggestions for improvement of the intervention.

#### Economic evaluation

The economic evaluation will be carried out using the Trimbos questionnaire for Costs associated with psychiatric illness (Tic-P) [49] and the Productivity and Disease Questionnaire (PRODISQ) [50]. Healthcare consumption is measured with two items of the Tic-P. The items are (1) in how far have participants made use of the healthcare facilities in the last four weeks? and (2) to what extent did they receive other help (e.g. from family and friends or homecare) in the last four weeks?. Participants can answer these questions with yes (1) or no (0). In case they made use of a healthcare facility (answer ‘yes’), they are asked how often they made use of it. Production losses due to illness and absenteeism will be measured with 6 items of the PRODISQ. Utilities will be derived from the EuroQol (EQ-5D) scores. Mean incremental cost per patient and the incremental cost utility ratio (ICER) will be calculated. The economic evaluation will be assessed from a societal perspective, thus including the intervention costs (such as costs for building the intervention, costs for hosting the website), the costs of formal and informal health care (TIC-P), and the economic costs due to productivity losses in paid and unpaid work in the four weeks preceding the trial (PRODISQ).

### Statistical analysis

The data of partners will be coded directly after being collected and will be entered into a statistical database to ensure accuracy and completeness of the data. Before we start with our data analysis, we will check if our data is normally distributed. If it is not, we will choose an non-parametric test. All data will be analyzed using SPSS version 20.

#### Descriptive statistics

A flow chart of participation during the total study will be drawn. Reasons for drop-out will be summarized. Percentages of missing values and dropout will be displayed. Background variables and summarized scores on questionnaires as mentioned in chapter 6 will be given. Basic psychometric analyses will be conducted to verify scale structure and internal consistency of the used questionnaires.

#### Effects

One-way ANOVA’s and χ^2^-tests will be performed to see if there are no significant differences at baseline between the two conditions for any of the demographic variables or outcome measures. Non-significant differences will indicate successful randomization. Intention-to-treat analyses will be conducted with use of SPSS missing values analysis to impute all missing data on the continuous measuring multiple imputation methods in SPSS. To examine differences between the conditions on all outcome measures, ANOVA (group x time) will be used. In the case of significant group x time – interactions, Tukey’s post hoc tests will be used.

Effect sizes on the primary outcome variable (HADS total) at post-intervention will be calculated with Cohen’s D using the means and pooled standard deviations of the measurements of the conditions (effect size of above .56 are considered large, .33-.55 are considered moderate, and less than .33 are considered as small [51].

#### Moderation and mediation analysis

The moderating effect of the socio-demographics (age, gender, education, working situation, family situation) and disease-related characteristics of the patients (type of cancer, stage of disease, duration and treatment of cancer) on the effectiveness of the intervention will be analyzed using regression analyses.

Mediation analysis will be performed as described by Preacher and Hayes [52,53]. Aim of this analysis is to assess whether psychological flexibility, self-compassion, supportive behavior, posttraumatic growth and resilience are mediators in the effect between the independent variable and the dependent variables.

### Sample size calculation

Based upon a previous, similar study [54] we expect effect sizes of at least d = 0.5. To demonstrate the presence of an effect of at least d = 0.5 as statistically significant in a two-tailed test at alpha = 0.05 and a power of (1-beta) = 0.80, a minimum of 64 participants in each condition will be required at follow-up (power calculation in G*Power). We have extended our sample size with 5 participants per condition in order to take normal distribution of the data as well as possible post hoc tests into account. Anticipating a drop-out rate of 20% between T0-measurement and T3-measurement, at least 87 participants per condition need to be included at T0-measurement. The total study cohort comprises thus 261 participants.

## Discussion

*Hold on, for each other* is the first web-based self-help intervention for partners of cancer patients that is based on both a clear theoretical framework and an iterative and user-centered development. The main purpose of the RCT is to evaluate the (cost-)effectiveness of *Hold on, for each other*, a recently developed web-based intervention for partners of cancer patients. Additional goals are: (1) to examine if psychological flexibility, self-compassion, mastery, supportive behavior, posttraumatic growth and resilience are mediators of the intervention’s effects on the partners’ mental health; (2) to examine the moderating effects of the socio demographics (age, gender, education, working situation, family situation) and disease-related characteristics of the patients (sort of cancer, stage of disease, duration and treatment of cancer); and (3) to examine to what extend participants are satisfied with the intervention.

### Strengths and limitations of the intervention

*Hold on, for each other* is a unique product. This web-based intervention is based on a clear theoretical framework (ACT) and the needs and wishes of the end-users have been carefully taken into account by the use of co-creation. We think that it is this combination that can make *Hold on, for each other* a successful intervention for partners of cancer patients who are in need of support. Also, this intervention is unique because it is positively framed. Partners who participated in our needs assessments agreed that an intervention for partners of cancer patients should be based on a positive approach. According to them, an intervention should be a source of hope and energy and it should focus on things that still can be done, instead of things that no longer can be done (because of their partner’s disease). Therefore, the focus of the intervention is based on the concept of making the best of life in a difficult time.

To the best of our knowledge, *Hold on, for each other* is one of the first web-based interventions for partners of cancer patients. In a literature review only three other web-based interventions for caregivers of cancer patients were found [9]. As we described earlier, the internet and recent technologies offer various possibilities (availability, easy accessible, flexibility) that can be of great value for this target group. Most importantly, partners can do the intervention at any convenient time. Daily exercises are short and can often be done while working, doing the household or giving care.

Another strength of the intervention is that it consists of a variety of components. Partners of cancer patients are offered a package of different features including information, psychological exercises, peer support, practical tips and text messages. Partners can choose freely which of the components they want to use, and in which way they want to use them. For example they can decide if they want to have contact with peers, and if so, they can choose if they want to actively write down their own experiences or if they merely want to read experience of other peers. Also, persuasive elements (such as text messages, tunneling and social support) are incorporated in the design of intervention in order to improve adherence to the intervention [24].

If proven effective, *Hold on, for each other* may easily be implemented in the Dutch healthcare system. One part of our implementation plan is to inform the various stakeholders from the beginning of the project: partners and cancer patients via patient societies (e.g. NFK), the Dutch Cancer Society, health care professionals as physicians, nurses, psychologist, social workers and drop-in centers. We have already started informing the stakeholders in the context of recruitment of participants. If the intervention is found to be effective, we expect that hospitals and other organizations with a focus on psycho-oncology (such as drop-in centers, general practitioners, patient organizations) will be interested in referring partners of cancer patients to this easy accessible psychosocial care option. Health care is rapidly changing and incorporating all kinds of e-health applications. In general, there is a growing interest in web-based, automated screening and monitoring of physical and psychological functioning of patients and partners as part of general portals with different functions (information, electronic dossiers, email, et cetera).

*Hold on, for each other* may also have some limitations. First of all not every partner may be interested in a web-based self-help intervention. We realize that some partners (for example elderly people) might prefer face-to-face contact with a health care professional instead of a (web-based) self-help intervention. However, we believe that the most important step is that partners of cancer patients are at least offered any kind of help. After that, they can decide for themselves if they need help, and whether they would prefer face-to-face contact with a health care professional or whether they would like to participate in a (web-based) self-help intervention. Besides, we think that it doesn’t have to be one or the other. Face-to-face consultation and web-based support can also become more blended, in order to fully utilize the possibilities and advantages of both forms of support. This may also be an appropriate solution for people with severe distress for whom a mere self-help intervention is not sufficient. Highly distressed partners could participate in *Hold on, for each other* under supervision of a health care professional (e.g. a psychologist). The professional could guide them through the intervention and he or she could check on them and constantly monitor on the partners’ (mental) health.

Another limitation may be that the intervention is not targeting bereavement. Therefore, *Hold on, for each other* is not applicable for partners who have already lost their ill spouse.

### Strengths and limitations of the RCT

Our study will answer questions regarding the (cost-)effectiveness of *Hold on, for each other* and possible determinants of the effects of the intervention on psychological distress in partners of cancer patients. We will also conduct a detailed process evaluation to obtain insight in processes in use of the intervention (e.g. time spent on the website, number of exercises completed, content of the messages exchanges). Additionally, satisfaction with the intervention will be measured. For example, participants will be asked if the intervention met their expectations, if they liked the intervention and what they thought about the content of the feedback. These insights can help us to improve the intervention. Furthermore, long-term effects will be studied and an economic evaluation will be done. These two aspects are also highly relevant for a successful implementation in the Dutch healthcare system.

Our study also has some limitations. First, we have no long-term data for the waiting list control condition. For ethical reasons, the participants in this group receive the intervention after the T1 measurement (three months after the baseline measurement). Second, we expect that it is highly challenging to find enough partners of cancer patients who are willing to participate in this trial. From other studies among informal caregivers of cancer patients it is known that it is difficult to find enough participants to meet the previous calculated power (e.g. [55-57]). In order to anticipate on this challenge, we make use of a variety of recruitment channels and recruitment strategies (see Table 3).

To conclude, this study will yield valuable knowledge about the (cost-)effectiveness of a newly developed web-based self-help intervention for partners of cancer patients. If proven to be effective, *Hold on, for each other* may be offered as standard service for partners of cancer patients in the healthcare system.
